# Identification of Factors on Blood Selenium Levels in the US Adults: A Cross-Sectional Study

**DOI:** 10.3390/nu16111734

**Published:** 2024-06-01

**Authors:** Ya-Zhi Bai, Yi-Xiong Gao, Shuang-Qing Zhang

**Affiliations:** National Institute for Nutrition and Health, Chinese Center for Disease Control and Prevention, 27 Nanwei Road, Beijing 100050, China; yzbaichinacdc@163.com (Y.-Z.B.); gaoyx@ninh.chinacdc.cn (Y.-X.G.)

**Keywords:** Se intake, blood Se, factors, American adults, National Health and Nutrition Examination Survey

## Abstract

Selenium (Se) is an essential trace element for humans and its low or high concentration in vivo is associated with the high risk of many diseases. It is important to identify influential factors of Se status. The present study aimed to explore the association between several factors (Se intake, gender, age, race, education, body mass index (BMI), income, smoking and alcohol status) and blood Se concentration using the National Health and Nutrition Examination Survey 2017–2020 data. Demographic characteristics, physical examination, health interviews and diets were compared among quartiles of blood Se concentration using the Rao-Scott χ^2^ test. Se levels were compared between the different groups of factors studied, measuring the strength of their association. A total of 6205 participants were finally included. The normal reference ranges of blood Se concentration were 142.3 (2.5th percentile) and 240.8 μg/L (97.5th percentile), respectively. The mean values of dietary Se intake, total Se intake and blood Se concentration of the participants were 111.5 μg/day, 122.7 μg/day and 188.7 μg/L, respectively, indicating they were in the normal range. Total Se intake was the most important contributor of blood Se concentration. Gender, race, education status, income, BMI, smoking and alcohol status were associated with blood Se concentration.

## 1. Introduction

Selenium (Se) as a crucial trace mineral for humans exerts substantial biological functions, such as the endoplasmic reticulum homeostasis, immune response, regulation of transcription factors and apoptosis, control of the cellular redox state and development of the central nervous system through selenoproteins [1]. Humans obtain Se mainly through foods and supplements, whereby the Se contents depend on the varied soil Se contents. Approximately 80% of the population in the world are deficient in Se (less than 55 μg/day) due to insufficient Se consumption [2].

Unfortunately, owing to a narrow safe range of Se, low or high Se status is found to be associated with the increased high risk of many diseases. Low Se levels increase the risk of Keshan disease, cretinism, immune dysfunction and cognitive impairment, whereas high Se levels elevate the occurrence of cancer, type 2 diabetes mellitus, neurological diseases such as amyotrophic lateral sclerosis, and endocrine diseases [3]. In addition, excessive Se intake or selenosis leads to some acute reactions, including garlic odor and metallic taste in mouth, hair or nail loss, nausea, diarrhea, skin rashes and irritability [4]. Because blood Se concentration is recognized as the reflection of Se intake [5], it is important to identify influential factors of Se status for the maintenance of a safe blood Se range to decrease those risks. 

To our knowledge, there are few studies to simultaneously evaluate the effects of several factors (Se intake, gender, age, race, education, body mass index (BMI), income, smoking and alcohol status) on blood Se. Based on the controllability and practical significance of most of these factors, i.e., some factors are influenced by self-development and lifestyles of participants, we hypothesize that blood Se levels in the US adult population are normal and that there is an association between blood Se and diet, gender, age, race, socioeconomic status, BMI and lifestyle. Therefore, the present study aimed to explore the association between those factors and blood Se concentration among American adults.

## 2. Materials and Methods

### 2.1. Study Design and Participants

The national cross-sectional National Health and Nutrition Examination Survey (NHANES) conducted by the US Centers for Disease Control and Prevention National Center for Health Statistics (NCHS) used a complex multistage sampling method to obtain representative samples with a combination of interviews and examinations for the assessment of the health and nutritional status of children and adults in the USA. All the participants provided informed consent. Initially, questionnaires including demographic, health-related history and cigarette use were carried out among the participants aged 18 years or older via household interview by trained interviewers. After the at-home interview, the participants meeting all the inclusion criteria were invited to continue participation in the survey by coming to a mobile examination center (MEC) two to four weeks later. At the MEC, the first dietary recall interview was collected in-person followed by the alcohol data collection, and the second interview was collected by telephone 3 to 10 days later. During the MEC, body measure data and blood samples were collected for further testing.

**Figure 1:**
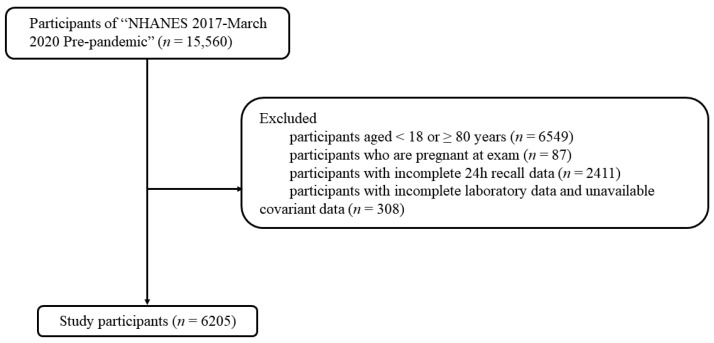
The selection process of study participants.

The National Health and Nutrition Examination Survey (NHANES) suspended field operations due to the coronavirus disease 2019 pandemic, resulting in an incomplete cycle for the NHANES 2019–2020; therefore, the data of the NHANES 2019–2020 must be combined with NHANES 2017–2018 for analysis. The present study was based on the NHANES 2017–March 2020 with a total of 15,560 individuals, and following exclusion criteria as shown in Figure 1, 6205 participants were finally included. The NHANES was carried out in accordance with the principles of the Declaration of Helsinki and its ethics was approved by the Institutional Review Committee of the NCHS. Due to the present study being based on the secondary analysis, it did not need additional ethics approval.

Gender, age and race were self-reported demographic information. Among these, race was categorized into Mexican American, other Hispanic, Non-Hispanic White, Non-Hispanic Black and other race. Education was defined by the question “What is the highest grade or level of school completed or the highest degree received?” and categorized into three levels: <high school, high school and >high school. The ratio of family income to poverty (PIR, <1.3 (low), 1.3–4.0 (medium), >4.0 (high)) [6] was a measure of household income and calculated by dividing total annual family (or individual) income by the poverty guidelines specific to the survey year. The lower PIR, the poorer participants [6]. The BMI data were calculated as weight in kilograms divided by height in meters squared and classified <18.5 (underweight), 18.5–25.0 (normal weight), 25.0–30.0 (overweight), ≥30.0 (obese). Smoking status was assessed via the question “Do you now smoke cigarettes?” and grouped as every day, somedays and never. Alcohol information was based on the self-report of the participants to the question “Ever have 4/5 or more drinks every day?” (yes/no).

### 2.2. Dietary and Supplemental Se Intakes, and Blood Se Concentration

Dietary data were acquired via two 24 h recalls. Total Se intake consisted of dietary and supplemental Se intakes obtained by averaging two respective 24 h recall data. Inadequate and excessive Se intakes were defined as less than the recommended dietary allowance (RDA) of 55 μg Se/day and more than the tolerable upper intake level of 400 μg Se/day for adults, respectively [7]. Blood Se concentration was detected by triple quadrupole inductively coupled plasma mass spectrometry. According to the previous study [4], the normal ranges of blood Se concentration were defined as the 2.5th and 97.5th percentiles for the overall US population. More experimental method details were described in the NHANES web at https://www.cdc.gov/nchs/nhanes/index.htm (accessed on 1 November 2021).

### 2.3. Statistical Analysis

Blood Se concentration was divided into quartiles based on the weighted population distribution. Demographic characteristics, physical examination, health interviews and diets were compared among quartiles of blood Se concentration using the Rao-Scott χ^2^ test. The LSD (least significant difference) test was used to compare the differences in Se intake and Se level between different groups of factors. Weighted linear regression was conducted to evaluate the association between several factors (Se intake, gender, age, race, education, BMI, PIR, smoking and alcohol status) and blood Se concentration. A *p* < 0.05 was considered statistically significant. Statistical analyses were conducted by SAS 9.4 version (SAS Institute Inc., Cary, NC, USA).

## 3. Results

### 3.1. Population Characteristics for Blood Se Concentration

Table 1 shows characteristics of the population categorized by quartiles of blood Se concentration. The percentage values of males and females were 48.7% and 51.3%, respectively. The age of the participants varied from 18 to 79 years old. More than half the participants had a degree of more than high school level. Additionally, the percentage values of the participants with hypertension, diabetes and stroke history were 31.0%, 11.0% and 3.5%, respectively. The weighted mean values of dietary Se intake, total Se intake and blood Se concentration of participants were 111.5 μg/day, 122.7 μg/day and 188.7 μg/L, respectively. The numbers of the participants with inadequate and excessive Se intakes were 602 and 17, respectively. The normal reference ranges of blood Se concentration were 142.3 (2.5th percentile) and 240.8 μg/L (97.5th percentile), respectively. There were 155 (only 2%) persons with blood Se deficiency and 155 (also only 2%) participants with Se toxicity. The percentage of participants taking Se supplements was 18.7% (1162 participants). For gender, race and education, statistically significant differences were observed among quartiles of blood Se concentration. There was no significant difference for age, BMI, PIR, smoking status, alcohol status, dietary Se intake, total Se intake, hypertension history, diabetes history and stroke history between quartiles of blood Se concentration.

### 3.2. Comparison of Se Intake and Se Level between Different Groups of Factors

Table 2 shows the Se intake and Se level between different groups of factors. Males had higher Se intake and blood Se than females. The participants aged 40–59 years had higher Se intake, but no difference in blood concentration was found among the three age groups. As for race, Mexican American, Non-Hispanic Whites and other races had greater Se intakes than Non-Hispanic Blacks and Non-Hispanic Whites, and Non-Hispanic Blacks had lower Se levels compared to other races. Additionally, Non-Hispanic Whites had higher Se levels than Non-Hispanic Blacks. The participants with more than high school level education possessed higher Se intake and blood Se concentration than those with less than high school and equal to high school education. Blood Se level was higher in overweight participants than in underweight, normal and obese participants. The participants with the highest income possessed the highest Se intake and those with the lowest income presented with the lowest blood Se concentration. Smokers showed lower blood Se. Alcohol users had higher Se intakes but lower blood Se concentrations.

### 3.3. Association between Factors and Blood Se Concentration

The association between all the factors and blood Se concentration is presented in Table 2. Total Se intake was the most important determinant of blood Se concentration (Table 3). Males tended to have higher blood Se concentration than females. The participants with greater educational levels appeared to have higher blood Se concentrations. The Non-Hispanic Blacks had lower blood Se concentration. Smokers had lower blood Se concentrations than nonsmokers (Table 3).

## 4. Discussion

In this study, the NHANES 2017–2020 data were used to explore several factors affecting blood Se level. Males aged 40–59 years, some races (Mexican American, Non-Hispanic White and other race) and highest educated population had higher Se consumption. Overweight participants, highest educated Non-Hispanic White and Non-Hispanic Black had higher blood Se levels. Smokers and alcohol users showed lower Se levels.

The average total Se intake and blood Se concentration were 122.7 μg/day and 188.7 μg/L, respectively, which were lower than those (174 μg/day and 253 μg/L) in the American seleniferous areas [8]. However, Se intake and blood Se concentration in this study were higher than those reported in other countries worldwide [9,10], with Se intake of approximately 95% of the participants above 55 μg/day, suggesting sufficient soil Se content in the USA. The Se-rich foods include Brazil nuts, crab meat, shrimp, allium vegetables, brown rice, whole wheat bread and skimmed milk. Se intake and blood Se vary globally. In Finland, Se fertilization increased average dietary intake from 40.0 μg Se/day to 80.0 μg Se/day [10]. In the low Se area of China, mean daily Se intake was 8.8 μg/day, resulting in the very low serum Se (24 μg/L) in that population [9]. In the present study, only 17 participants (only 0.3%) had excessive Se intake (>400 μg/day), nevertheless, considering that excessive Se intake led to acute side effects and chronic Se exposure increased the risks of nervous system abnormalities, it was very vital to maintain a safe Se intake range to reduce Se toxicity.

In the present study, there was a significantly positive association between dietary Se/total Se intakes and blood Se concentration, especially, total Se was the most important contributor for blood Se. Similarly, a previous study conducted in the American seleniferous area also found a strong correlation between Se intake and blood Se concentration [8]. A dose-response analysis found a non-linear relationship between Se intake and plasma Se concentration, and the predicted coefficients below and above such a cut-off of 70.0 μg/day were 1.25 and 0.43, respectively [11]. The aforementioned different results might be attributed to the sample size and source, Se species in foods and supplements consumed by the participants (organic Se mainly in foods and inorganic Se mainly in supplements), Se bioavailability (organic Se > inorganic Se) and analysis methods [12]. Additionally, some metals such as lead and mercury could interact with Se [13,14,15]; unfortunately, the intake information for those metals were not available in the NHANES. 

In the present study, males had higher blood Se than females, which was consistent with the results of previous studies [6,16,17], partly due to the apparent sexual dimorphism related to sex hormones [18]. The trans-selenation pathway was regulated by sex hormones, suggesting selenomethionine metabolism and selenocysteine formation and the availability for selenoprotein synthesis are not the same in both sexes [18]. Given the sex difference in Se intake and blood Se concentration, establishing gender-based Se reference intakes should be considered in future Se RDA revisions [3]. 

In our study, age was not significantly associated with blood Se concentration, which coincided with the findings of other studies in the USA [8,19]. There was no significant difference in blood Se concentration among the three age groups (18–39, 40–59, 60–79), although the participants aged 40–59 years had higher Se intake than those aged 60–79. However, the results of some previous studies were inconsistent, i.e., decreased blood Se [20] with age and increased serum Se [21] with age, partly because of changed absorption and excretion efficiency. Elevated Se concentration was demonstrated to improve cognitive functions [6,22], which was beneficial to the amelioration of cognitive decline in the elderly.

In the USA, the mean blood Se concentration was higher in white subjects compared to black subjects, which might be attributed to different geographical areas with varied soil Se content, differences in food choices and genetic differences in Se pharmacokinetics [17]. The participants with the education status of more than high school possessed prominently higher blood Se concentration, which was in line with the findings of the NHANES 2011–2014 [6,23], possibly because they cared more about dietary quality and had access to Se-enriched foods to enhance Se intake. The participants with low income had lower blood Se concentration than those with high income, which was similar to the previous report [24]. Interestingly, in our study, Se intake was higher in the high-income participants than those low-income participants. Moreover, participants with underweight were found to have a lower blood Se level than overweight participants, partly due to their lesser Se intake. Our study showed higher blood Se concentration in overweight participants compared to that in obese participants, and the previous result found reduced serum Se level in obese female patients [25]. There was an inverse association between smoking and blood Se. Smokers, especially those smoking every day, had significantly lower Se intake than non-smokers, which was in agreement with previous findings in the USA [8]. Notably, drinkers with more Se intake showed lower blood Se concentration compared to non-drinkers with less Se intake, which was caused by changes in hepatic structure and function induced by alcohol [26]

The strengths of the present study included a large representative sample for the reduction in sampling error, high-quality NHANES datasets and the weighted liner regression because of a complex survey design. The results could direct health-related policy decisions in the field of health and disease in the future.

However, there were several limitations in the present study. Firstly, owing to NHANES data based on a cross-sectional study, dietary Se data were self-reported with two 24 h recalls with inevitable recall bias and it only reflected short-term Se intake not long-term Se intake status. Therefore, a longitudinal study might be reasonable. Additionally, dietary data plus occupational exposure data more truly evaluated Se level in the human body. Secondly, Se species in foods and supplements were not available in the NHANES 2017–2020. Thirdly, alcohol information in the present study was not an optimal reflection of alcohol consumption levels of the participants.

## 5. Conclusions

The present results showed that the majority of US adults were in the safe range of blood Se concentrations and few participants were at risk of selenosis. Taken together, Se intake was the most primary determinant of blood Se. Gender, race, education status, income, BMI, smoking and alcohol status were associated with blood Se concentration. Considering the effects of Se status on certain chronic diseases shown in epidemiological studies, this study can provide baseline information for future health-related research and revision of guidance values.

## Figures and Tables

**Table 1:** Study population characteristics categorized by blood Se concentration in the NHANES 2017–2020.

	Overall *n*	Blood Se Concentration (μg/L)				
	Weighted % (95% CI)	Q1 (85.15–169.34)	Q2 (169.35–183.94)	Q3 (183.95–200.76)	Q4 (200.77–526.40)	*p*-Value
Characteristic		*n*				
		Weighted% (95% CI)				
	6205	1551	1552	1551	1551	
		21.3 (18.3, 24.3)	24.4 (22.8, 25.9)	27.1 (24.4, 29.7)	27.2 (24.4, 30.0)	
Gender						<0.05
Male	2988	655	728	768	837	
	48.7 (46.3, 51.0)	19.2 (15.6, 22.7)	22.9 (20.2, 25.7)	26.5 (22.5, 30.5)	31.4 (27.7, 35.1)	
Female	3217	896	824	783	714	
	51.3 (49.0, 53.6)	23.4 (20.0, 26.8)	25.7 (23.2, 28.2)	27.6 (24.9, 30.3)	23.3 (20.0, 26.5)	
Age						0.14
18–39	2134	531	553	538	512	
	38.9 (36.0, 41.8)	19.9 (15.7, 24.2)	27.2 (23.7, 30.8)	26.7 (23.5, 29.8)	26.2 (23.1, 29.3)	
40–59	2118	492	523	553	512	
	34.8 (32.4, 37.1)	21.9 (17.8, 26.1)	21.5 (19.2, 23.9)	29.2 (25.7, 32.6)	26.2 (23.1, 29.3)	
60–79	1953	528	476	460	489	
	26.3 (23.1, 29.6)	22.6 (19.3, 25.9)	23.9 (20.3, 27.5)	24.9 (20.8, 29.0)	28.6 (23.6, 33.7)	
Race						<0.05
Mexican American	750	136	206	206	202	
	8.8 (6.1, 11.5)	17.1 (12.7, 21.5)	27.2 (22.1, 32.3)	27.0 (20.2, 33.9)	28.7 (22.9, 34.4)	
Other Hispanic	647	181	171	145	150	
	8.0 (6.3, 9.7)	27.4 (23.8, 31.1)	25.3 (20.5, 30.2)	26.5 (19.9, 33.2)	20.7 (15.9, 25.5)	
Non-Hispanic White	2121	481	512	587	541	
	61.8 (56.3, 67.4)	20.3 (16.3, 24.4)	24.1 (22.0, 26.1)	28.0 (23.9, 32.0)	27.6 (23.7, 31.5)	
Non-Hispanic Black	1734	562	467	358	347	
	11.4 (8.3, 14.4)	27.4 (23.0, 31.8)	27.0 (23.8, 30.1)	22.7 (19.4, 25.9)	23.0 (17.9, 28.0)	
Other Race	953	191	196	255	311	
	10.0 (8.1, 11.9)	19.5 (15.3, 23.7)	20.1 (15.0, 25.1)	26.8 (23.9, 29.8)	33.6 (28.0, 39.2)	
Education						<0.05
<High school	940	266	243	219	212	
	9.2 (8.0, 10.4)	28.0 (23.2, 32.9)	25.1 (20.4, 29.8)	24.1 (18.5, 29.6)	22.9 (17.2, 28.5)	
High school	1391	388	361	311	331	
	26.8 (24.3, 29.3)	24.3 (19.8, 28.8)	26.4 (22.4, 30.5)	23.0 (18.6, 27.4)	26.3 (21.8, 30.7)	
>High school	3577	841	863	950	923	
	64.0 (61.0, 67.0)	19.4 (15.9, 22.9)	23.3 (21.6, 25.0)	29.3 (26.4, 32.2)	28.0 (24.5, 31.6)	
PIR						0.22
<1.3	1585	485	385	380	335	
	19.7 (17.5, 21.8)	25.1 (21.5, 28.8)	21.9 (19.2, 24.6)	28.6 (24.9, 32.4)	24.3 (19.9, 28.7)	
1.3–4.0	2378	548	589	591	650	
	39.4 (35.7, 43.1)	20.1 (16.3, 23.9)	24.4 (21.8, 27.0)	26.3 (23.0, 29.6)	29.2 (25.1, 33.3)	
>4.0	1520	332	379	414	395	
	40.9 (37.2, 44.7)	20.1 (15.9, 24.3)	24.8 (21.7, 27.9)	28.1 (23.2, 33.0)	27.0 (22.6, 31,4)	
BMI						0.64
<18.5	93	29	25	10	19	
	1.3 (0.8, 1.8)	30.0 (15.2, 44.8)	28.3 (11.2, 45.4)	20.1 (6.7, 33.5)	21.7 (6.6, 36.7)	
18.5–25.0	1459	404	369	366	320	
	24.8 (22.5, 27.0)	23.1 (18.7, 27.5)	25.1 (21.9, 28.2)	26.5 (21.8, 31.2)	25.3 (21.1, 29.6)	
25.0–30.0	1863	410	461	463	529	
	31.4 (29.2, 33.7)	19.5 (15.8, 23.3)	24.7 (22.2, 27.3)	26.1 (20.5, 31.7)	29.6 (25.0, 34.3)	
≥30.0	2742	686	688	693	675	
	42.5 (39.5, 45.5)	21.2 (17.4, 24.9)	23.6 (21.6, 25.7)	28.4 (24.4, 32.3)	26.8 (23.2, 30.4)	
Smoking status						0.32
Every day	866	264	215	203	184	
	30.5 (27.6, 33.5)	24.9 (20.5, 29.2)	26.6 (21.5, 31.8)	26.1 (21.9, 30.4)	22.3 (16.8, 27.9)	
Somedays	260	73	71	57	59	
	10.1 (7.6, 12.6)	19.3 (10.4, 28.3)	32.2 (19.0, 45.4)	25.7 (16.0, 35.4)	22.7 (12.2, 33.2)	
Not at all	1384	328	334	343	379	
	59.3 (55.5, 63.2)	21.5 (16.7, 26.3)	23.7 (19.4, 28.0)	25.2 (21.1, 29.3)	29.6 (24.5, 34.7)	
Alcohol use						0.68
Yes	840	241	192	218	189	
	14.3 (12.6, 16.0)	21.9 (17.2, 26.6)	21.9 (17.9, 25.9)	28.9 (21.9, 35.8)	27.3 (20.6, 34.1)	
No	4672	1128	1202	1165	1177	
Dietary Se (μg/d)						0.52
6.70–73.84	1551	422	381	361	387	
	22.8 (20.8, 24.8)	20.5 (17.3, 23.7)	25.5 (22.8, 28.1)	27.7 (23.5, 31.9)	26.4 (22.6, 30.1)	
73.85–101.29	1551	417	384	386	364	
	25.4 (23.8, 27.0)	24.9 (21.0, 28.7)	24.3 (21.0, 27.6)	25.8 (20.7, 30.9)	25.0 (19.2, 30.9)	
101.30–135.74	1550	362	410	391	387	
	26.3 (24.5, 28.0)	19.0 (14.1, 23.9)	24.5 (21.6, 27.3)	28.4 (24.5, 32.2)	28.2 (23.1, 33.2)	
135.75–780.20	1553	350	377	413	413	
	25.6 (24.1, 27.0)	21.0 (16.4, 25.6)	23.4 (20.5, 26.3)	26.4 (22.0, 30.8)	29.2 (24.4, 34.0)	
Total Se (μg/d)						0.27
6.70–77.69	1549	422	398	350	379	
	22.2 (20.1, 24.3)	21,6 (17.5, 25.8)	26.3 (23.0, 29.6)	24.5 (20.8, 28.1)	27.6 (23.2, 31.9)	
77.70–107.64	1553	436	402	372	343	
	25.8 (24.1, 27.5)	24.4 (20.6, 28.2)	25.5 (21.0, 29.9)	26.6 (20.5, 32.8)	23.5 (17.7, 29.4)	
107.65–147.89	1551	377	386	386	402	
	25.8 (24.1, 27.5)	20.8 (15.0, 26.6)	24.5 (20.5, 28.6)	27.5 (22.9, 32.0)	27.2 (22.0, 32.3)	
147.90–830.20	1552	316	366	443	427	
	26.2 (24.3, 28.0)	18.6 (14.7, 22.5)	21.5 (18.9, 24.2)	29.3 (23.5, 35.1)	30.6 (25.9, 35.3)	
Hypertension history						
Yes	2252	583	546	535	588	0.42
	31.0 (28.5, 33.6)	21.6 (18.6, 24.6)	22.6 (19.7, 25.5)	26.5 (23.1, 30.0)	29.2 (25.1, 33.4)	
No	3947	966	1006	1015	960	
	68.9 (66.3, 71.5)	21.2 (17.6, 24.8)	25.2 (22.6, 27.8)	27.3 (24.1, 30.5)	26.3 (23.5, 29.1)	
Diabetes history						
Yes	883	235	202	196	250	0.23
	11.0 (10.1, 11.9)	22.9 (18.8, 26.9)	22.3 (16.4, 28.1)	23.4 (18.9, 28.0)	31.5 (25.6, 37.3)	
No	5144	1276	1306	1310	1252	
	86.7 (85.5, 87.9)	21.1 (18.0, 24.3)	24.7 (22.7, 26.7)	27.5 (24.6, 30.3)	26.7 (23.7, 29.7)	
Stroke history						
Yes	272	92	61	59	60	0.38
	3.5 (2.8, 4.3)	24.5 (16.6, 32.3)	19.3 (13.2, 25.3)	30.9 (25.6, 36.2)	25.3 (16.3, 34.4)	
No	5630	1402	1404	1419	1405	
	96.4 (95.6, 97.1)	21.4 (18.3, 24.5)	24.5 (22.8, 26.1)	26.9 (24.3, 29.6)	27.2 (24.2, 30.1)	

*p* value was tested by Rao-Scott χ^2^ test. BMI: body mass index. PIR: poverty income ratio. Se: selenium.

**Table 2:** Comparison of Se intake and Se level between different groups of factors in the NHANES 2017–2020.

Characteristic	Se Intake (μg/d)	Differences	Blood Se Concentration (μg/L)	Differences
Gender				
Male	140.1 (137.6, 142.6)	M > F (*p* < 0.0001)	188.5 (187.5, 189.4)	M > F (*p* < 0.0001)
Female	101.1 (99.5, 102.8)		184.2 (183.3, 185.1)	
Age				
18–39	119.1 (116.5, 121.7)		185.5 (184.4, 186.5)	
40–59	122.5 (119.7, 125.2)	40–59 > 60–79 (*p* < 0.05)	186.9 (185.8, 187.9)	
60–79	118.0 (115.2, 120.8)		186.5 (185.1, 187.8)	
Race				
Mexican American	125.3 (121.0, 129.6)	Mexican American > Other Hispanic (*p* < 0.05)	189.0 (187.2, 190.8)	Other Race > Non-Hispanic White > Other Hispanic > Non-Hispanic Black (*p* < 0.05)
Other Hispanic	116.4 (111.5, 121.2)	Mexican American > Non-Hispanic Black (*p* < 0.05)	183.8 (181.9, 185.7)	Mexican American > Other Hispanic (*p* < 0.05)
Non-Hispanic White	122.1 (119.4, 124.8)	Other Hispanic < Non-Hispanic White (*p* < 0.05)	188.1 (186.9, 189.3)	
Non-Hispanic Black	113.5 (110.7, 116.3)	Other Hispanic< Other Race (*p* < 0.05)	180.9 (179.7, 182.0)	
Other Race	124.9 (120.7, 129.0)	Non-Hispanic White> Non-Hispanic Black (*p* < 0.05)	191.5 (189.8, 193.1)	
		Non-Hispanic Black < Other Race (*p* < 0.05)		
Education				
<High school	111.2 (107.6, 114.9)	“<High school” < “>High school”	183.8 (182.2, 185.4)	“<High school” < “>High school”
High school	116.1 (112.8, 119.4)	“High school“ < “>High school”	184.4 (183.0, 185.8)	“High school“ < “>High school”
>High school	124.4 (122.3, 126.5)		187.5 (186.6, 188.4)	
BMI				
Underweight	117.2 (104.5, 130.0)	Overweight > Obese (*p* < 0.05)	181.4 (175.5, 187.2)	Underweight < Overweight (*p* < 0.05)
Normal weight	121.2 (117.9, 124.4)		184.4 (183.1, 185.6)	Normal weight < Overweight (*p* < 0.05)
Overweight	121.7 (118.8, 124.7)		188.8 (187.6, 190.1)	Overweight > Obese (*p* < 0.05)
Obese	117.9 (115.6, 120.2)		185.9 (184.9, 186.9)	
PIR				
Low	111.4 (108.5, 114.2)	Low < Medium < High (*p* < 0.05)	182.7 (181.5, 184.0)	Low < Medium (*p* < 0.05)
Medium	120.3 (117.8, 122.7)		187.7 (186.6, 188.3)	Low < High (*p* < 0.05)
High	128.2 (124.7, 131.6)		188.4 (187.0, 189.7)	
Smoking status				
Every day	115.2 (111.2, 119.2)	Every day < Somedays (*p* < 0.05)	182.1 (180.4, 183.7)	Every day < Not at all (*p* < 0.05)
Somedays	127.5 (119.0, 135.9)	Every day < Not at all (*p* < 0.05)	183.3 (180.3, 186.2)	Somedays< Not at all (*p* < 0.05)
Not at all	125.6 (122.1, 129.1)		187.8 (186.4, 189.3)	
Alcohol use				
Yes	131.9 (127.4, 136.4)	Yes > No (*p* < 0.0001)	183.9 (182.0, 185.7)	Yes < No (*p* < 0.05)
No	119.6 (117.9, 121.4)		186.5 (185.7, 187.2)	

BMI: body mass index. PIR: poverty income ratio. Se: selenium.

**Table 3:** Weighted linear regression analyses of the association between several factors (Se intake, gender, age, race, education, BMI, income, smoking and alcohol status) and blood Se concentration in the NHANES 2017–2020.

Factors	*β* (95% CI)	*p*-Value
Dietary Se (μg/day)	0.03 (0.01, 0.04)	<0.05
Total Se (μg/day)	0.05 (0.04, 0.07)	<0.001
Female (vs. male)	−2.66 (−5.26, −0.06)	<0.05
Age (y)	0.03 (−0.06, 0.11)	0.52
Race (vs. other race)		
Mexican American	−1.14 (−5.64, 3.36)	0.61
Other Hispanic	−6.59 (−11.2, −1.97)	<0.05
Non-Hispanic White	−1.98 (−5.16, 1.20)	0.21
Non-Hispanic Black	−6.65 (−10.8, 2.51)	<0.05
Educational status (vs. <high school)		
High school	−0.51 (−3.93, 2.91)	0.76
>High school	2.73 (−0.94, 6.40)	0.14
PIR (vs. low)		
Medium	1.49 (−0.84, 3.83)	0.20
High	0.07 (−2.56, 2.70)	0.96
BMI (vs. underweight)		
Normal weight	5.76 (−0.65, 12.17)	0.08
Overweight	8.52 (2.25, 14.79)	<0.05
Obese	7.33 (1.17, 13.49)	<0.05
Smoking status (vs. not at all)		
Everyday	−2.65 (−6.12, 0.81)	0.13
Somedays	−3.44 (−9.54, 2.66)	0.26
Alcohol use (vs. yes)	1.59 (−0.97, 4.16)	0.21

BMI: body mass index. PIR: poverty income ratio. Se: selenium.

## Data Availability

The datasets generated and analyzed in the present study are available on the website of NHANES datasets 2017–2020 (https://www.cdc.gov/nchs/nhanes/index.htm (accessed in 2022)).
